# “Manifesto” for Advancing the Control and Elimination of Neglected Tropical Diseases

**DOI:** 10.1371/journal.pntd.0000718

**Published:** 2010-05-25

**Authors:** Peter J. Hotez, Bernard Pecoul

**Affiliations:** 1 Department of Microbiology, Immunology, and Tropical Medicine, George Washington University and Sabin Vaccine Institute, Washington, D. C., United States of America; 2 DNDi (Drugs for Neglected Diseases Initiative), Geneva, Switzerland

Neglected tropical diseases (NTDs) are the most common infections of the world's poorest people and the leading causes of chronic disability and poverty in low- and middle-income countries [1]–[3]. NTDs (Table 1) especially affect children and young women of reproductive age [4], and consequently deprive them of their health and economic potential [3]. NTDs also impair agricultural productivity and are an important reason why the world's poorest 1.4 billion people who live below the poverty line cannot escape destitution and despair [3]. Despite the devastating effect of these diseases on health and development, with evidence that their global burden is as great as that of any other serious disease [1]–[3], financial support for control and elimination efforts, as well as research and development (R&D), have been inadequate [2], [5]. Indeed, in Millennium Development Goal 6 (to “combat HIV/AIDS, malaria and other diseases”), NTDs were not even specifically mentioned but merely considered as part of the “other diseases” [6]. However, policy makers are slowly beginning to appreciate the importance of NTDs.

**Table 1:** Neglected tropical diseases.

Category	Infections
Helminth Infections	Ascariasis Trichuriasis Hookworm Strongyloidiasis Toxocariasis and larva migrans Lymphatic filariasis OnchocerciasisLoiasisDracunculiasisSchistosomiasisFood-borne trematodiasesTaeniasis-cysticercosisEchinococcosis
Protozoan Infections	LeishmaniasisChagas disease Human African trypanosomiasisAmebiasisGiardiasisBalantidiasisToxoplasmosisTrichomoniasis
Bacterial Infections	BartonellosisBovine tuberculosisBuruli ulcerCholeraEnteric pathogens (*Shigella*, *Salmonella*, *E. coli*)LeprosyLeptospirosisRelapsing feverTrachomaTreponematoses: Bejel, pinta, syphilis, yaws
Viral Infections	Dengue feverJapanese encephalitisJungle yellow feverOther arboviral infectionsRabiesRift Valley feverViral hemorrhagic fevers
Fungal Infections	MycetomaParacoccidiomycosis
Ectoparasitic Infections	ScabiesMyiasisTungiasis

Modified from http://www.plosntds.org.

The World Health Organization (WHO) has a new Department of Neglected Tropical Diseases, and WHO-TDR (Special Programme for Research and Training in Tropical Diseases) has a new 10-year strategic plan with support from UN agencies, member states, and private philanthropies. At the same time, funding for integrated NTD preventive chemotherapy control from the governments of the US and UK has increased dramatically and is approaching US$100 million annually, while support remains strong for product development partnerships from the Bill & Melinda Gates Foundation, Médecins Sans Frontières (MSF), and a few European governments. Recently, the new Director of the US National Institutes of Health, Francis Collins, has targeted NTDs as a research priority, and the UK charity Wellcome Trust has agreed with the multinational pharmaceutical company Merck & Co. to allocate substantial funds for a joint, not-for-profit research center in India to develop inexpensive “antipoverty” vaccines against neglected diseases [7], [8]. Additional efforts to combat NTDs are also being shared among major multinational pharmaceutical companies (i.e., Novartis, GlaxoSmithKline, Pfizer, Sanofi-Aventis, Merck & Co.) and others who have also committed resources and made investments in research and development for these conditions. Thus, although at present only about 10% of the global funds required for preventive chemotherapy and NTD mass drug administration have been committed, and although R&D for NTDs has not even reached the so-called 10/90 gap [9], (meaning only 10% of available global R&D spending is committed for diseases that disproportionately affect 90% of the world living in low-income and middle-income countries), there is cautious optimism that such disparities could diminish in the coming decade.

With a combination of funds from the group of eight (G8) nations, emerging economies (e.g., Brazil, India), multinational companies, and private philanthropic sources, together with a community of scientists, physicians, and other healthcare workers, global public health experts and policy makers committed to NTDs have begun to deliberate about how future resources and investments should be best allocated, particularly in terms of an appropriate balance between implementation and R&D. The leadership of key international agencies such as WHO, ministries of health in disease-endemic countries, and the communities themselves is key to achieve any ambitious strategy. With a global dialogue now underway, this is an appropriate time to present an eight-point manifesto (“a public declaration of motives and intentions by a government or by a person or group regarded as having some public importance” [2], [11]) for NTDs.

## 1. All NTDs are “tool ready”

**Figure 1:**
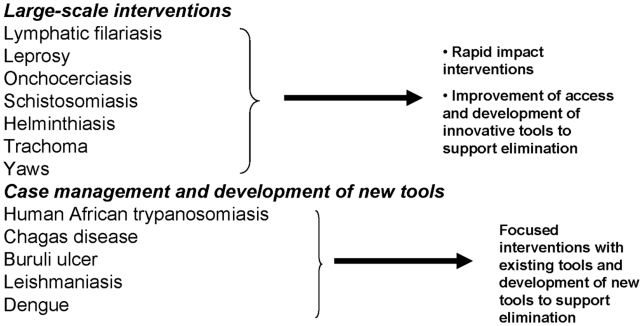
What is needed to combat NTDs?

Tools refer partly to the drugs used to treat NTDs in low- and middle-income countries, particularly when these are used as agents of control and elimination through mass drug administration [12]. Today, most of the NTDs have tools that could be implemented now, even if for some diseases such tools are far from being perfect or complete (Figure 1). For example, each year, hundreds of millions of poor people receive donated or low-cost generic drugs, which in some epidemiological environments have led to the elimination of lymphatic filariasis (LF), onchocerciasis, and trachoma [13]–[15], as well as reduction of morbidity for the three major soil-transmitted helminth infections (i.e., ascariasis, trichuriasis, and hookworm infection), and for schistosomiasis (Table 2) [1]–[3], . At present, populations at risk for LF and onchocerciasis are receiving the highest global drug coverage (>40%), whereas less than 10% of school-aged children at risk for soil-transmitted helminth infections and schistosomiasis are receiving treatment [12]. To improve global coverage rates, in many cases the control of these large-scale–intervention NTDs could be achieved by simultaneous administration of several drugs, sometimes in a so-called “rapid impact package” costing around US$0.50 per person per year [1]–[3], [12], [16], [17]. Similarly, leprosy has been eliminated in many countries through multi-drug therapy [18]. The NTD manifesto mandates that mass drug administration programs continue to expand until they reach the entire “bottom billion” who deserves access to essential medicines. Simultaneously, support must be provided for parallel operational research to optimize integration of the different NTD mass treatment programs and for other aspects of implementation science.

**Table 2:** Control strategies, challenges, research need and major recent advances for selected NTDs.

Disease	Control Strategy	Challenges	Research Needs	Major Recent Advances
Chagas disease	• Interruption of transmission through vector control and improved blood transfusion	• Control of non-domicile vectors; s• Sustained vector control• Millions infected at risk of disease	• Strategies for control of non-domicile vectors• Better drugs and diagnostics	• Pediatric benznidazole could be available soon• New compounds in development
Dengue	• Active surveillance and case management• Selective vector control	• Poor mosquito control• Increase in man-made risk factors• Case management in epidemics	• Better methods for mosquito control• Better tools: vaccines, drugs, case management	• Vaccines in development
Human African trypanosomiasis (HAT)	• Active surveillance, case finding and treatment• Selective vector control	• Poor surveillance• Poor diagnostics• Toxic drugs	• Better tools: drugs and diagnostics	• Development of simplified HAT treatment: NECT• Fexinidazole in development stage
Leishmaniasis	• Case finding and treatment• Selective vector/animal reservoir control, elimination in the Indian subcontinent	• Long, difficult, expensive treatment• Practical limitations of diagnostics• Low priority (cutaneous leishmaniasis)• Poor health systems	• Better tools: drugs and diagnostics• Better case-finding and treatment strategies• Anti-leishmania vaccine	• Paromomycin, miltefosine, liposomal amphotericin B• Combination therapies• Vaccine in development
Leprosy	• Case-finding and multi-drug treatment	• Incomplete multi-drug treatment coverage• Integrating/sustaining control• Impact on transmission not known	• Integration of leprosy control• Improved diagnosis of infection• Simplified multi-drug treatment regimen• Possible BCG vaccination strategies	• Elimination achieved in many countries• Re-evaluation of elimination targets
Lymphatic filariasis	• Interruption of transmission through periodic mass treatment• Disability alleviation by local hygiene	• Elimination target by 2020• Limited effect of current drugs• Co-endemicity (loa loa, onchocerciasis)	• Shorten duration of control measures• Drugs that kill/sterilize adult worms (macrofilaricide)• New detection methods	• Elimination of transmission in several countries• Ivermectin donation (Merck) and albendazole (GSK)• Some antibiotics (tetracycline, rifampicin) found effective
Onchocerciasis	• Periodic mass treatment to eliminate the disease as a public health problem	• Need to sustain high coverage• Eradication not possible with current tools• Limited effect of current drug• Over-reliance on one single drug• Co-endemicity (loa loa)	• Drugs that kill/sterilize adult worms (macrofilaricide)• Shorten duration of control measures• New detection methods• Resistance markers	• Ivermectin donation (Merck)• Some antibiotics (tetracycline, rifampicin) found effective• Moxidectin in development stage• Control in ten west African countries• No new cases of blindness due to onchocerciasis in the Americas in the past decade
Soil-transmitted Helminth Infections	• Morbidity control through periodic mass treatment	• WHO target to treat >75% school-age children at risk• Inclusion of pre-school children (<5 y)• Low cure rates with single dose• Over-reliance on one single drug	• Operational research to integrate with other NTD control efforts and to improve coverage• Better drugs or combination of drugs• Better control measures• Resistance markers• Antihelminthic vaccines to prevent re-infection and forestall drug resistance	• New antihelminthic drugs• Human hookworm vaccine in development
Schistosomiasis	• Morbidity control through periodic treatment in high-risk populations	• WHO target to treat >75% school-age children at risk• Limited availability of praziquantel• Over-reliance on one single drug	• Operational research to integrate with other NTD control efforts and to improve coverage• Better drugs or combinations• Resistance markers• Antihelminthic vaccines to prevent re-infection and forestall drug resistance	• Antimalarial drugs found effective• New drug candidates• Decreased prevalence in some countries• Partial donation of praziquantel (Merck KGaA)• At least two vaccines in development
Trachoma	• SAFE (surgery, antibiotics, face washing, environmental control) strategy	• Global elimination of trachoma by the year 2020• Over-reliance on one single drug	• Operational research to integrate with other NTD control efforts and to improve coverage	• Elimination in selected countries

Tools for NTDs also refer to field-based diagnostics and vector-control strategies (in some cases using geographic information systems and remote sensing), as well as improvements in water and sanitation. The nearly-complete eradication of dracunculiasis is an outstanding example of how non-drug-based approaches can achieve sustained control [19]. There have been successes in the local control of dengue and other arboviral infections through mosquito control measures [20], and in trachoma elimination through a combined strategy of surgery, antibiotics, face washing, and environmental control (SAFE strategy) [1], [3], [12], [21].

At present, we can also achieve substantial sustainable control for the important vector-borne kinetoplastid NTDs (i.e., human African trypanosomiasis [HAT], Chagas disease, and leishmaniasis). For example, during the early part of the 20th century, Jamot and his colleagues implemented mobile teams for Gambian HAT in West Africa. These health teams, with the logistical support of the military, traveled to endemic areas to identify human cases for treatment with either tryparasamide or, later, pentamidine, together with a vertically structured vector-control strategy (i.e., for tse-tse only) [2], [22]. For stage 1 HAT, this approach using pentamidine is still valid today, while for stage 2 HAT (affecting the central nervous system) a new available treatment is nifurtimox–eflornithine combination therapy (NECT), which reduces the time and cost required for treatment with eflornithine alone and is safer and more effective than previous arsenical treatment options [23]. Similarly, new cases of Chagas disease have been eliminated in some South American countries through detection of the bug vectors and insecticide spraying for vector control, in addition to programs of diagnostics and treatment with benznidazole or nifurtimox, providing medical care to patients, and screening blood donors [24], [25]. Finally, an elimination program of visceral leishmaniasis has been launched on the Indian subcontinent through passive and active case detection, early diagnosis and treatment, integrated vector management (including indoor residual spraying and insecticide-treated bed nets) and vector surveillance, as well as environmental management and social mobilization [26]–[29].

The NTD manifesto mandates that such programs of case detection, treatment, and integrated vector management should also continue to receive adequate support. Health education is yet another important element for prevention, and, for some NTDs, it is the only available tool (i.e. the food-borne trematode infection opisthorchiasis [30] and Buruli ulcer [31]). Indeed, with a few possible exceptions, we now have control tools in hand for almost all major NTDs, but their use must be expanded and, where appropriate, improved strategies for their use must continue to be developed [32], and supported through a robust program of operational research and implementation science.

## 2. All NTDs are “tool deficient”

Although tools exist to control, or in some cases even eliminate, NTDs, for many of these diseases the tools and implementation strategies available are suboptimal, incomplete, or inadequate to sustain elimination efforts. Consequently, substantial investments in R&D are urgently needed to develop new-generation control tools and strategies for their improved use and implementation.

The currently available drugs for HAT are highly toxic or need long treatment regimens and careful patient monitoring, which are often difficult in resource-poor settings or fragile health systems located in conflict or post-conflict endemic areas [22], [23], [33]. NECT is an efficacious and easier to administer alternative compared to arsenicals or eflornithine alone [23], but it is only a temporary suboptimal solution and better tools are still needed to achieve HAT elimination. The completed genome for African trypanosomes and other kinetoplastids offers great potential for the development of new drugs [34]. However, there is still a big gap between genomics data and target identification and validation, and subsequent compound screening. Several years will be needed to develop screening hits that become drug candidates through the lead optimization process. To respond to the urgent needs of new, better, and inexpensive treatments for HAT, several product development partnerships and WHO-TDR have initiated a systematic search for drug targets and drugs candidates from existing compounds made by various pharmaceutical organizations and research institutes [35]. One of the compounds is fexinidazole [36], which has been now been taken all the way from discovery and into clinical development. However, because of the high attrition rate in drug development, continued efforts in building the HAT drug pipeline need to be maintained until new oral drugs are available.

Similarly for Chagas disease, the two existing drugs, benznidazole and nifurtimox, have several limitations in terms of safety, questionable efficacy in the prevention of long-term complications associated with cardiomyopathy and megacolon/megaesophagus, and difficult delivery in fragile healthcare systems in the poorest regions of Latin America [37], [38]. In addition, most patients identified through systematic surveillance are children, and pediatric formulations do not exist, although a nascent program to develop a pediatric formulation of benznidazole is underway. Based on genomics and proteomics analyses, some new and promising approaches exist for the development of drugs for Chagas disease, including new agents that target ergosterol and trypanothione biosynthesis, farnesyl-pyrophosphate synthase, purine salvage pathways, and a unique cysteine protease known as cruzipain [37], [38]. As with HAT, a systematic search for drug candidates from the pipelines of pharmaceutical companies has been conducted and several antifungal azoles have been identified as possible clinical candidates for the treatment of Chagas disease. However, because of the lack of clearly defined efficacy endpoints and no predictive animal model, drug development for Chagas disease is a very challenging task.

For visceral leishmaniasis, the existing tools still largely depend on antimonials, which have not yet been optimized to reduce toxicity and prevent emerging drug resistance [28], [29],. Furthermore, only three new effective treatments have been licensed over the past decade, and even these remain largely inaccessible to most control programs of leishmaniasis in resource-poor settings (Table 2) [39]. Although several combination treatments are under development to prevent the emergence of drug resistance and to reduce treatment duration, these are not going to be enough for disease elimination. Therefore, the NTD manifesto mandates urgent action to provide adequate support for the development of such anti-kinetoplastid drugs. Vaccines for all three kinetoplastid infections are also in early-stage development, and a recombinant leishmaniasis vaccine is in clinical testing [7], [8], [40], [41]. Similarly, vaccines for other nonhelminthic NTDs such as amebiasis and the neglected mycobacterial infections Buruli ulcer and leprosy are in early development [7], [8], [42]–[44], and at least two live attenuated tetravalent vaccine candidates for dengue fever are in phase 2 clinical trials, with numerous other vaccine candidates also under development [7], [8], [45], [46].

For the soil-transmitted helminth infections (the world's most common NTDs), albendazole is still the only agent available that can treat all three major infections (i.e., ascariasis, trichuriasis, and hookworm infection) when used as a single dose in mass drug administration campaigns. Although mebendazole can still be used for ascariasis, a recent meta-analysis has shown that single-dose mebendazole has high failure rates against hookworm [47]. This finding means that we must rely on a single drug to treat more than one billion infected people every year, despite the fact that this class of benzimidazole anthelminthic is highly susceptible to drug resistance when widely used to deworm livestock [48]. Therefore, development of new anthelminthics, such as the amino-acetonitrile derivatives that are highly effective as veterinary agents [48], or tribendimidine, a nicotinic acetylcholine receptor agonist discovered in China [49], is urgently needed. Alternatively, mebendazole or albendazole could be combined with other existing anthelminthic drugs (i.e., pyrantel or levamisole) to reduce development of drug resistance, and a new *Bacillus thuringiensis* crystal protein is showing promise in preclinical testing [50]. A human hookworm vaccine is under product and clinical development and would be used in a program of vaccine-linked chemotherapy to prevent hookworm reinfection after treatment [7], [8], [51]. Similarly, for schistosomiasis praziquantel is the only available agent to treat more than 200 million people, and while drug resistance has not been clearly shown, development of new drugs through automated screening [52], [53], or by mining the genome [54] is urgently needed. Anti-schistosome vaccines together with chemotherapy are an important new option [7], [8], [55], and there is a need to think about how to integrate such new tools into changing demographic, health, and social systems [56]. For both onchocerciasis and LF, if a macrofilaricide was available (i.e., a drug for mass distribution that destroys the adult worm), as opposed to the existing microfilaricidal drugs ivermectin and diethylcarbamazine citrate, fewer rounds of annual distribution would be necessary and elimination efforts would be made much more efficient [57]. Antibiotics that destroy the parasite's bacterial symbionts are also being explored for this purpose [58].

For most of the major NTDs, the current approaches to diagnosis and case detection were developed in the early- or mid-twentieth century. There is an urgent need to develop new diagnostics and rapidly introduce them into ongoing and future control programs [59], [60].

As the US and UK Governments increase funding for integrated NTD control, there is an urgent need to also increase significantly R&D efforts developed by product development partnerships and other organizations. Indeed, the poorest people living in low- and middle-income countries have the right to access not only essential medicines but also innovation. Unfortunately, global initiatives, especially from the G8 nations, have largely lacked efforts to support R&D. The George Institute has recently analyzed how much money is invested every year on R&D for neglected diseases [5]. About three-quarters of total neglected disease R&D annual spending is for HIV/AIDS, malaria, and tuberculosis, leaving only about US$600 million worldwide for all NTDs per year, with only US$139 million for all kinetoplastid infections, US$132 million for diarrheal diseases, US$127 million for dengue, US$67 million for all human helminth infections, and less than US$10 million for each neglected mycobacterial infection, trachoma, and Buruli ulcer [5]. Because of the huge disease burden from these infections, such modest R&D support reflects what may be a 1/99 gap relative to other chronic diseases in developed nations. We also need to ensure funds are made available for clinical research (which is expensive) into new drugs and vaccines. The development of truly modern antimicrobial/antiparasitic agents and vaccines will take many years and is likely to remain a high-risk endeavor with respect to the level of investment in R&D and the high attrition rate of drug discovery. In order to facilitate this research, and due to the pressing needs in NTDs, governments and regulators need to ensure that incentives and enabling regulatory systems are made available to product developers [61].

## 3. All NTDs are “most neglected”

Because of the great disease burden of NTDs and the absence of adequate funding to support their control or elimination, each of the major NTDs listed in Table 1 should be considered as severely neglected. In contrast, diseases such as malaria and tuberculosis have been also neglected but they have received significantly more attention during the past ten years from the international community, with the creation of the US President's Emergency Plan for AIDS Relief, the US President's Malaria Initiative, the Global Fund to Fight AIDS, Tuberculosis, and Malaria, and considerable R&D investments from the Bill & Melinda Gates Foundation, NIH, Wellcome Trust, and at least five dedicated product development partnerships for drugs and vaccines to combat these conditions [5]. Funding for NTD control and R&D should be brought closer to the level of current support for HIV/AIDS, malaria, and tuberculosis.

## 4. There is a profound human rights dimension to NTDs

Although poverty is surely one of the main risk factors for neglected diseases, increasing evidence indicates an association between their prevalence and conflict and violation of human rights [62]. As noted above, NTDs affect the poorest of the poor, who have no economic and political power and are very often neglected by their governments. Many NTDs are disfiguring, causing severe social consequences. Most affected populations live in remote areas with limited or no access to treatment or prevention. Indigenous or aboriginal people are also disproportionately affected by NTDs [63], while in the Americas NTDs were introduced through the Atlantic slave trade and to this day they disproportionately affect non-white people [64], [65]. NTDs are found wherever extreme poverty occurs—not only in developing countries but in poor areas in developed countries including the USA and Europe [64], [66]. Mahatma Gandhi (who himself suffered from hookworm infection [67]) once said that a “civilization is judged by the treatment of its minorities” [68]. This observation is particularly relevant for people living with neglected diseases, which generally can be either treated or prevented at low cost.

## 5. NTDs destabilize societies and contribute to conflict

Many poor societies have either been recently engaged in a civil or international conflict or are currently at war [69]. The potentially destablilizing effects of NTDs, especially on agricultural productivity and food security, may partly explain why considerable geographic overlap has been observed between NTDs and recent conflict, especially for HAT, leishmaniasis, and onchocerciasis in sub-Saharan Africa [67], [70]. These conditions are likely to substantially contribute to conflict in low-income countries [62], [67], [70]. At a community level, the disease burden may destabilize a settlement to a point that entire villages are abandoned. Conflict areas are insecure and unstable, frequently with no functioning national disease program. In these situations, medical humanitarian assistance and innovative health strategies are greatly needed to combat NTDs [71].

## 6. Involvement by the WHO and other international health agencies is crucial for current and future NTD control

The community working on NTDs greatly appreciates the active involvement of the WHO, through their new Department of Neglected Tropical Diseases, WHO-TDR, and the regional offices. The technical advisory role and convening power of the WHO and their regional offices, and their active contributions to global control and elimination efforts, indicate success and commitment. Accordingly, WHO is absolutely essential for the future global control and elimination efforts supported by governments and private partners (NGOs, pharmaceutical companies, and philanthropic organizations). At the same time, it needs to be recognized that WHO is not alone in this success. UNICEF (United Nations International Children's Emergency Fund), UNDP (United Nations Development Program), FAO (Food and Agricultural Organization), the World Bank and several regional banks, as well as the NTD control public–private partnerships, have greatly contributed to global NTD control and elimination, and their ongoing efforts should be both applauded and encouraged.

## 7. Building health systems under the leadership of health ministries in disease-endemic countries and the communities is a high priority

Nothing is more important to the success of global NTD control than the involvement of communities themselves, with disease-endemic countries' health ministries providing leadership. Community-directed treatments for ivermectin, for instance, have helped the establishment of a key health system for onchocerciasis control [72], [73]. This and similar activities account for much of the high-level coverage for onchocerciasis and LF [73], and are vital for ensuring that in the near future treatment coverage for soil-transmitted helminth infections, schistosomiasis, and other NTDs reaches similar levels. In many areas of conflict and postconflict in Africa, community involvement in NTD control is one of the few actively functioning health systems. Such activities have facilitated the delivery of additional interventions such as insecticide-treated bed nets, antimalarial drugs, micronutrients, and childhood immunizations [74]. The coordination and leadership by health ministries is crucial to achieve sustainable control and elimination efforts for NTDs in integrating the different vertical strategies into a coordinated, strengthened public health system. To this end, NTDs need to be prioritized at the level of health ministries. This can occur with greater awareness and improved funding mechanisms for local control programs.

## 8. Moving forward through a global strategy combining access and innovation

Millennium Development Goal 8 (“develop a global partnership for development”) advocates for international partnerships to achieve all millennium targets [6]. Under the leadership of international organizations (WHO and its regional offices, UNICEF, FAO), all stakeholders—health ministries in disease-endemic countries, affected communities, public–private partnerships, research communities in both endemic and nonendemic countries, product development partnerships for the development of new tools, large and small nongovernmental organizations—should establish a well-functioning international strategy for NTD control. Global partnerships for NTDs are involved in the delivery of existing treatments and in the development of new ones. With adequate support from the G8 Governments and some emerging economies [75], notably through the G20, the international NTD community could substantially reduce poverty and serve as a highly efficient vehicle for millennium targets, and all this potentially at costs far lower than other international initiatives. Countries affected by NTDs must also assume responsibility in addressing the dire health needs of impoverished populations and work to deliver new policies that will develop health innovation capacity through research networks and technology transfer schemes.

Although NTDs threaten the lives of millions in the developing world, their burden on global health is under-recognized, often sidelined, and under-resourced.

Actions are urgently needed to promote interactions among scientists working on NTDs, to facilitate the dissemination of information about NTDs, to identify funding opportunities and the most cost-effective ways to fight NTDs, and to explore possibilities for international collaborations for promoting and implementing R&D projects. By highlighting important challenges in the fight against NTDs, this manifesto calls on the global community for urgent, renewed, and innovative efforts.
