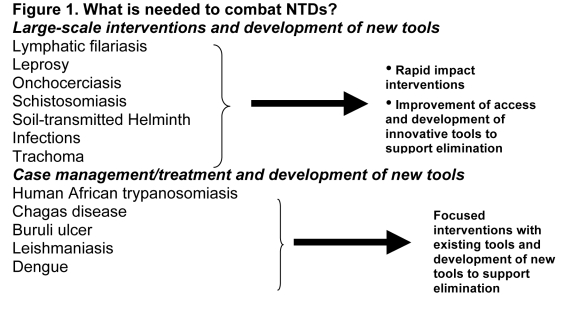# Correction: “Manifesto” for Advancing the Control and Elimination of Neglected Tropical Diseases

**DOI:** 10.1371/annotation/53d95072-6329-412d-a5e4-c1a00acd1934

**Published:** 2010-05-26

**Authors:** Peter J. Hotez, Bernard Pecoul

In Figure 1, part of the text was missing. Please see the corrected figure: 

**Figure:**